# Phytochemical Analysis and In-Vitro Biological Activities of Three Wild *Eryngium* Species: *E. beecheyanum, E. heterophyllum*, and *E. mexiae*

**DOI:** 10.3390/molecules30214250

**Published:** 2025-10-31

**Authors:** Mariana Villa-Santiago, Brenda Hildeliza Camacho-Díaz, Argelia López-Bonilla, Hortencia Gabriela Mena-Violante, Jeanette Guadalupe Cárdenas-Valdovinos, Zaida Ochoa-Cruz, María Valentina Angoa-Pérez

**Affiliations:** 1 Instituto Politécnico Nacional, Department of Research, CIIDIR IPN Unidad Michoacán, Jiquilpan 59510, Michoacán, Mexico; mvillas1900@alumno.ipn.mx (M.V.-S.); hmena@ipn.mx (H.G.M.-V.); jcardenasv@ipn.mx (J.G.C.-V.); zochoac1900@alumno.ipn.mx (Z.O.-C.); 2 CEPROBI–IPN, Instituto Politécnico Nacional, Carretera Yautepec-Jojutla s/n-km 85, Col. San Isidro, Yautepec 62739, Morelos, Mexico; bcamacho@ipn.mx (B.H.C.-D.); alopezb@ipn.mx (A.L.-B.)

**Keywords:** wild plants, medicinal plants, flavonoids, phenolic compounds, hierba del sapo

## Abstract

The genus *Eryngium* (Apiaceae Lindley) includes over 250 species distributed worldwide. In Michoacán, Mexico, 22 species have been recorded, among them *E. beecheyanum* (EB), *E. heterophyllum* (EH), and *E. mexiae* (EM), which are commonly used in traditional medicine. However, our understanding of their biology and chemical composition remains limited. This study evaluated the phytochemical profile, as well as the antioxidant and hypoglycemic activities of leaves and roots from these three wild species. Flavonoids, phenolic compounds, and sterols were analyzed using high-performance thin-layer chromatography (HPTLC). Antioxidant activity was assessed in vitro using ABTS·+ and DPPH· assays, while antihyperglycemic activity was determined by α-glucosidase inhibition. Six metabolites were detected across all species, with organ-dependent variation. In the leaves, EB showed a high rutin content (241.3 µg/mL), EM contained catechin (137.3 µg/mL), and EH exhibited β sitosterol (315.9 µg/mL). Both leaves and roots of all species showed notable antioxidant activity. EB leaves exhibited inhibition rates of 69.5% and 85.5% in ABTS•+ and DPPH• assays, respectively (IC_50_ = 22 and 23.47 µg/mL). EH roots showed higher activity, reaching 89.4% and 78.2% inhibition (IC_50_ = 21.8 and 20.72 µg/mL). Conversely, EM organs exhibited relatively lower radical scavenging capacities; however, EM leaves showed the highest α-glucosidase inhibition (49.1%). Overall, these results suggest that roots generally possess stronger antioxidant potential than leaves, whereas EM leaves stand out for their enzymatic inhibitory activity. These findings highlight the diverse phytochemical and bioactive profiles of *E. beecheyanum*, *E. heterophyllum*, and *E. mexiae*.

## 1. Introduction

The use of medicinal plants plays a significant role in human daily life, as they contribute not only to public health but also to social, cultural, and economic aspects [1,2]. The World Health Organization (WHO) reports that 80% of the global population relies on traditional medicine, with 85% of these individuals utilizing plants as part of their therapies [3]. Mexico is not the exception, being a country renowned for its ancestral traditions and extensive use of plants with therapeutic potential. It is estimated that 3000 to 5000 medicinal plant species are registered in the country; however, only about 1% have been scientifically investigated to elucidate their therapeutic properties [4,5]. The scarcity of comprehensive data hinders the effective management and sustainable utilization of medicinal plant resources [5]. Among these plants, the wild species belonging to the genus *Eryngium* L. stand out. This genus is part of the Apiaceae Lindley family, with more than 250 species identified worldwide to date [6]. In Mexico, an estimated 60 species have been recorded, 22 of which are reported in the state of Michoacán, including *E. beecheyanum* (EB), *E. heterophyllum* (EH), and *E. mexiae* (EM) [4]. Due to their distinctive morphology and diverse metabolic profiles, several *Eryngium* species have long been used traditionally to treat a wide range of ailments, including diabetes, renal disorders, fever, hypertension, gastrointestinal conditions, asthma, urinary tract infections, malaria, and infertility. Notably, the roots are commonly employed in folk medicine to relieve inflammatory conditions, headaches, sinusitis, urinary infections or inflammation, and snakebites [7,8,9].

Phytochemical analyses have shown that species of the *Eryngium* genus are rich in secondary metabolites such as phenolic compounds, flavonoids, tannins, saponins, terpenoids, coumarins, essential oils, polyacetylenes, and steroids [6,10]. These compounds perform multiple functions in different plant parts, and their synthesis depends on tissue localization, since each organ has specific metabolic requirements [11]. Moreover, environmental stressors—both biotic and abiotic—affect their production, contributing to the plants’ adaptation to adverse conditions [11,12].

Phenolic acids are widespread in plant tissues and are recognized for diverse biological activities [13]. Notable examples include caffeic acid (C_9_H_8_O_4_, molecular weight 180.16 g/mol), gallic acid (C_7_H_6_O_5_, molecular weight 170.12 g/mol), and ferulic acid (C_10_H_10_O_4_, molecular weight 194.18 g/mol); all of which are classified as polyphenols. all are classified as polyphenols and have reported antioxidant, antibacterial, anti-inflammatory, and antidiabetic properties [14,15,16]. Similarly, flavonoids participate in processes such as signaling, auxin transport and pigmentation [11,17], and exhibit antioxidant, antimutagenic, and anti-inflammatory activities that contribute to various pharmacological effects [18,19]. Rutin (C_27_H_30_O_16_, molecular weight 610.52 g/mol) and catechin (C_15_H_14_O_6_, molecular weight 290.27 g/mol) are widespread flavonoids whose roles in antioxidant, anti-inflammatory, and antidiabetic activities have been documented [17,20,21].

Phytosterols are plant sterols that play essential roles in plant physiology and possess various biological properties [22,23]. Among them, β sitosterol (C29H50O, MW 414.71 g·mol−1) is a prominent bioactive phytosterol found in plant cell membranes and has been associated with antioxidant, antimicrobial, anti-inflammatory, and antidiabetic effects [24,25]. Thus, flavonoids, phenolic compounds, and phytosterols not only perform specific functions in different plant organs but also confer healing properties by contributing to pharmacological activities of therapeutic interest [1,26]. Previous studies have identified several *Eryngium* species with medicinal potential, notably *E. maritimum* L., *E. planum* L., *E. dichotomum* Desf., *E. campestre* L., *E. creticum* Lam., *E. foetidum*, *E. palmatum*, and *E. caucasicum*, which have been extensively investigated [6,7,27]. Noriega-Cisneros et al. [28] reported that the ethanolic extract of *E. carlinae* exhibits hypolipidemic activity and may serve as an adjuvant in diabetes treatment. *E. billardierei* has been shown to reduce lipid accumulation, triacylglycerol content, and the enzymatic activity of glycerol 3 phosphate dehydrogenase (GPDH) in mature adipocytes [29]. Despite this potential, only a limited number of species have been studied to establish the efficacy and safety of their traditional uses. In the state of Michoacán, two species—hereafter referred to as EB and EH—are known locally as “hierba del sapo” and are integrated into regional cultural and traditional medicine, while EM is not used by inhabitants of the same areas. Nevertheless, given reports of bioactive metabolites in other *Eryngium* species, EM was considered worthy of study. All three species remain poorly investigated. Therefore, this study aimed to evaluate the phytochemical profiles and the antioxidant and antihyperglycemic activities of leaf and root extracts from the three wild species to identify their metabolites and assess their potential for traditional therapeutic use.

## 2. Results

### 2.1. Phytochemical Analysis of the EB, EH, and EM

**Figure 1:**
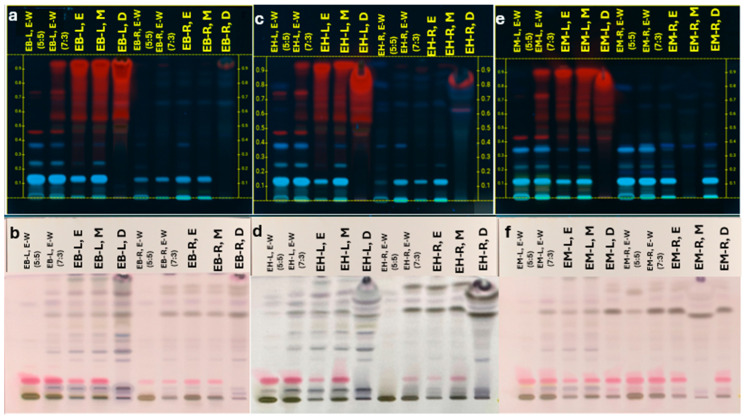
Visual HPTLC chromatograms showing metabolites in leaves and roots of *Eryngium beecheyanum* (EB), *E. heterophyllum* (EH), and *E. mexiae* (EM). EB-L/EB-R = leaf/root extracts of *E. beecheyanum*; EH-L/EH-R = leaf/root extracts of *E. heterophyllum*; EM-L/EM-R = leaf/root extracts of *E. mexiae*. Solvents: E = ethanol, M = methanol, W = water, D = dichloromethane. Mobile phase: toluene–ethyl acetate–formic acid (6:4:0.3 *v*/*v*/*v*). Panels (**a**,**c**,**e**): UV 365 nm, visualized with natural product reagent; panels (**b**,**d**,**f**): visible light, visualized with anisaldehyde.

**Figure 2:**
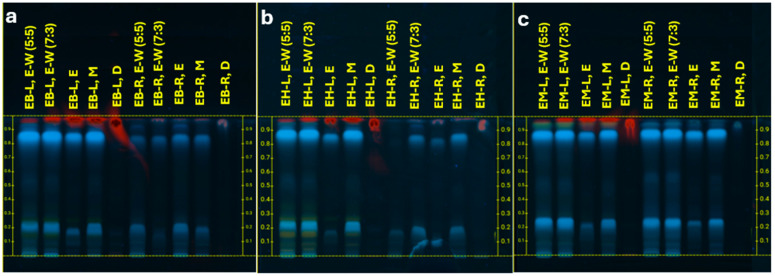
Visual HPTLC chromatograms showing metabolites in leaves and roots of *Eryngium* species. (**a**) *E. beecheyanum* (EB), (**b**) *E. heterophyllum* (EH); (**c**) *E. mexiae* (EM). EB-L/EB-R = leaf/root extracts of *E. beecheyanum*; EH-L/EH-R = leaf/root extracts of *E. heterophyllum*; EM-L/EM-R = leaf/root extracts of *E. mexiae*. Solvents: E = ethanol, M = methanol, W = water, D = dichloromethane. Mobile phase: ethyl acetate–acetic acid–water (8:1:1 *v*/*v*/*v*). Visualization: natural product reagent; image captured under UV 365 nm.

Phytochemical screening of leaf and root extracts from EB, EH, and EM revealed groups of compounds, including saponins, flavonoids, phenolic acids, and phytosterols (Figure 1 and Figure 2). Phenolics were visualized as blue to dark blue bands, flavonoids as yellow to orange bands, and saponins as violet pink bands, consistent with Thiem et al. [30] for other *Eryngium* species (*E. planum*, *E. campestre*, *E. maritimum*). Most metabolites were more effectively extracted with water–ethanol (3:7 *v*/*v*), whereas β sitosterol showed higher recovery with dichloromethane.

### 2.2. Quantification of Rutin in EB, EH, and EM

The presence of rutin (Rf = 0.16) was detected by HPTLC in leaf extracts of EB and EH (Table 1), with concentrations of 241.3 µg/mL (EB) and 189.1 µg/mL (EH); rutin was not detected in EM leaves nor in any root extracts. Scanner profiles correlated with the rutin standard, yielding correlation coefficients of 0.96 (EB) and 0.98 (EH).

### 2.3. Quantification of Catechin in EB, EH, and EM

Catechin (Rf = 0.15) was identified in both leaf and root extracts of all three species (Table 1). Leaf concentrations were highest in EM (137.3 µg/mL). Root concentrations were highest in EM (64.4 µg/mL) and EB (61.3 µg/mL). All species showed greater catechin levels in leaves than in roots. Correlation coefficients with the catechin standard were 0.97 for EB and EH, and 0.96 for EM, indicating strong agreement.

### 2.4. Quantification of Ferulic Acid in EB, EH, and EM

Ferulic acid (Rf = 0.51) quantification for the three *Eryngium* species are shown in Table 2. No significant differences (*p* ≤ 0.05) were observed between EH and EM leaf concentrations (105.5 and 108.7 µg/mL, respectively). Ferulic acid was not quantifiable in root extracts of any species. Correlation coefficients with the ferulic acid standard were 0.99 for all three species.

### 2.5. Quantification of Gallic Acid in EB, EH, and EM

Quantification of gallic acid (Rf = 0.20) is summarized in Table 2. Gallic acid was detected in all three species, with no significant differences (*p* ≤ 0.05) among leaf extracts of EB, EH, and EM. In roots, gallic acid was quantifiable only in EM (11.3 µg/mL). Similarity with the gallic acid standard was 0.99, 0.97, and 0.98 for EB, EH, and EM, respectively.

### 2.6. Quantification of Caffeic Acid in EB, EH, and EM

Quantification of caffeic acid (Rf = 0.40) is reported in Table 2. The highest leaf concentrations were found in EB and EM (71.3 and 68.5 µg/mL, respectively). In roots, EM showed the highest caffeic acid level (105.3 µg/mL). Correlation coefficients with the caffeic acid standard were 0.99 for EB and EM and 0.97 for EH (both leaves and roots).

### 2.7. Quantification of β-Sitosterol in EB, EH, and EM

β Sitosterol (Rf = 0.72) was detected in all three *Eryngium* species (Table 3). Leaf concentration was highest in EH (315.9 µg/mL), whereas root concentration was highest in EB (394.7 µg/mL). Similarity with the β sitosterol standard was 0.98 for leaf and root extracts of EB, EH, and EM.

**Figure 3:**
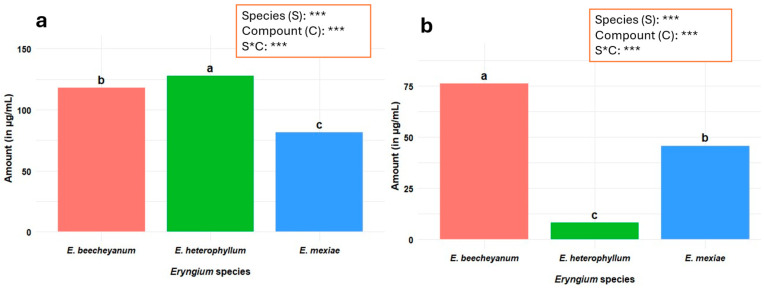
Comparison of metabolite content among *Eryngium* species. (**a**) Comparison among leaf extracts; (**b**) Comparison among root extracts. A two-way factorial analysis was performed considering *Eryngium* species (3) and compounds (6). S: *Eryngium* species; C: analyzed compounds; S × C: interaction effect between species and compounds. Significance levels are indicated as follows: *p* ≤ 0.001; ***. Different letters within a column indicate significant differences between treatments (Tukey’s test, *p* ≤ 0.05).

The comparison among the three *Eryngium* species based on the presence and quantity of identified metabolites is shown in Figure 3. In leaf extracts, EH exhibited the highest overall content and concentration (127.5 µg/mL). In root extracts, EB showed the highest metabolite content (73.0 µg/mL). Concentrations differed significantly across species, compounds, and their interaction (*p* ≤ 0.001).

### 2.8. Antioxidant Activity 

#### Antioxidant Activity Based on ABTS●+ and DPPH• Assays

All extracts from the three *Eryngium* species exhibited antioxidant activity. In leaf extracts (Table 4), ABTS•+ activity was highest in EB (220.0 µM TE/g DW) and EM (215.0 µM TE/g DW), while DPPH• activity was highest in EB (101.2 µM TE/g DW). IC_50_ values ranged from 19 to 23 µM TE/g DW.

The highest antioxidant activity in root extracts (Table 5) was observed in EH (266 µM TE/g DW) by the ABTS•+ assay. EH and EM also showed notable DPPH• activity. IC_50_ values ranged from 20 to 24 µg/mL.

### 2.9. Hypoglycemic Activity of EB, EH, and EM Extracts

All extracts from the three *Eryngium* species inhibited α-glucosidase activity (Table 6). The highest inhibition in leaf extracts was observed for EM (80.4%), close to the positive control. In root extracts, no significant differences were observed among species. IC_50_ values for EB and EH leaf extracts were > 7 µg/mL, while the other treatments had IC_50_ < 6 µg/mL.

### 2.10. Pearson Correlation

**Figure 4:**
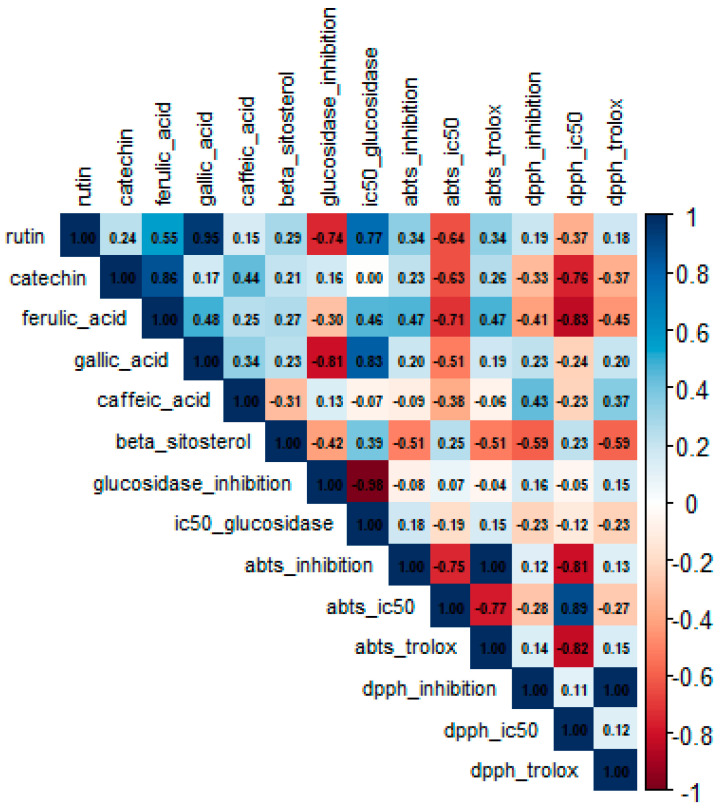
Heatmap of Pearson correlation coefficients between metabolite contents (rutin, catechin, ferulic acid, gallic acid, caffeic acid, β sitosterol) and biological activity parameters for ethanol–water (7:3 *v*/*v*) leaf and root extracts of *E. beecheyanum* (EB), *E. heterophyllum* (EH), and *E. mexiae* (EM).

Pearson correlation analysis revealed significant associations between quantified metabolites and the antioxidant and antihyperglycemic activities in the *Eryngium* species (Figure 4). Strong positive correlations were observed between rutin and gallic acid (r = 0.95) and between catechin and ferulic acid (r = 0.86). Rutin and gallic acid also correlated positively with α-glucosidase IC_50_ (r = 0.77 and r = 0.83, respectively).

## 3. Discussion

Six metabolites were identified at notably high concentrations in three underexplored *Eryngium* species (EB, EH, EM), with flavonoids among the predominant compounds consistent with previous reports for the genus. Rutin in EB and EH leaves was quantified at 180–242 μg/mL, substantially higher than values reported for other *Eryngium* species—approximately 2–4 times greater than *E. kotschyi* (63.0 µg/mg) [8], and 3–5 times greater than whole-plant *E. campestre* (49.1 μg/mL) [31]. Catechin was detected in leaves (80–140 μg/mL) and roots (40–65 μg/mL) of all three species, concentrations 2–9 times higher than methanolic whole plant *E. campestre* extracts (16.2 μg/mL) [31], and 3–6 times higher than *E. maritimum* roots (12.3 μg/mL), but 1–3 times lower than catechin reported in leaves of the same species (195 μg/mL) [32].

Among the phenolic compounds identified in EB, EH, and EM, ferulic acid was detected in the leaves of all three species at concentrations ranging from 70 to 110 μg/mL. These levels were approximately 380–600 times higher than those reported in *E. maritimum* (0.18 μg/mL) [32], 1–2 times greater than those observed in *E. serbicum* (59.6 μg/mL) [6] (also in leaf extracts), and 5–8 times higher than those reported for *E. campestre* (12.8 μg/mL) in whole-plant extracts [31]. Caffeic acid was identified in both tissues of all evaluated species (leaves: 35–70 μg/mL; roots: 7–105 μg/mL). These concentrations were 1.3–14 times higher than those reported for *E. campestre* (5.4 μg/mL) [31], and were similar to or slightly higher than those observed in *E. kotschyi* (55.1 μg/mL) [8] and *E. serbicum* (83.9 μg/mL in leaves; 64.3 μg/mL in roots) [6]. Gallic acid was quantified in all three species, both in leaves (30–33 μg/mL) and in roots (≈11 μg/mL). These concentrations were 1.2–4 times higher than those previously reported for *E. campestre* in whole-plant extracts (9.1 μg/mL) [31], suggesting greater accumulation of this compound in the studied species. However, the levels detected in roots were 2–6 times lower than those reported for *E. maritimum* (67.5 μg/mL) [32].

Regarding phytosterols, β sitosterol was detected in all three analyzed *Eryngium* species. In leaves, concentrations ranged from 173.9 to 315.9 μg/mL, and in roots from 93.1 to 394.7 μg/mL. The presence of this metabolite has also been reported in other species, such as *E. carlinae* [33]; in aerial parts and roots of *E. thorifolium* [34], and in *E. foetidum* [35].

Collectively, the three analyzed species (EB, EH, and EM) contained rutin, catechin, ferulic acid, caffeic acid, gallic acid, and β sitosterol at variable concentrations depending on species and organ (leaf and root). Most of the observed concentrations exceeded those reported for other *Eryngium* species, indicating substantial potential for traditional medicinal use. The organ-specific distribution of these compounds suggests functional accumulation related to the physiological and ecological demands of each tissue [11]. This differential localization not only reflects tissue-specific metabolic specialization but may also guide the therapeutic use of extracts prepared from particular plant organs.

Concerning biological activities in the genus *Eryngium*, antioxidant activity has been reported for ethanolic leaf and root extracts of *E. serbicum* [6]. In leaves, IC_50_ values of 21.1 μg/mL (DPPH•) and 34.1 μg/mL (ABTS•+) were recorded; in roots, values were 25.6 μg/mL (DPPH•) and 35.2 μg/mL (ABTS•+). Compared with the afore mentioned antioxidant activity, the activity observed in EB, EH, and EM extracts was lower, suggesting greater antioxidant capacity in those referenced extracts. Conversely, the ethanolic extract of *E. foetidum* showed considerably lower antioxidant activity in DPPH and ABTS•+ assays, with IC_50_ values of 6222.8 ± 95.3 μg/mL and 6761.3 ± 141.6 μg/mL, respectively [36]. These results underscore the efficacy of EB, EH, and EM extracts relative to other species in the genus. It should be noted that IC_50_ is inversely proportional to antioxidant activity: the lower the IC_50_, the higher the antioxidant potential of the extract.

Overall, extracts from the three evaluated *Eryngium* species demonstrated significant antioxidant activity, indicating the presence of bioactive compounds with high potential, such as flavonoids and phenolic acids, which are known for their free-radical scavenging capacity. Among these, rutin (IC50 = 23.3 μg/mL in DPPH• and 43.5 μg/mL in ABTS•+) and gallic acid (IC_50_ = 2.0 μg/mL in DPPH• and 1.6 μg/mL in ABTS•+), reported by [6] and detected by HPTLC in the EB, EH, and EM extracts analyzed in this study, are notable. Flavonoids exert antioxidant activity primarily by donating electrons or hydrogen atoms, enabling neutralization of reactive oxygen species (ROS) such as superoxide (O_2_−^·^) and peroxynitrite (ONOO−). This capacity is attributed to hydroxyl groups in their structure, which react with free radicals to form more stable, less reactive compounds. Additionally, some flavonoids, including rutin and epicatechin, can inhibit xanthine oxidase, thereby reducing endogenous ROS generation and contributing to protection against oxidative cellular damage [6,37]. Phenolic compounds, in turn, exert antioxidant effects mainly by donating hydrogen atoms to reactive species such as DPPH, ABTS●+, ·O_2_−, ·OH, and ONOO−, thus neutralizing free radicals and preventing oxidative damage. Some phenolics, such as gallic acid, can also chelate metal ions (e.g., Fe^2+^), blocking Fenton reactions and suppressing lipid peroxidation, thereby reducing oxidative cellular damage. They have additionally been shown to activate the Nrf2/HO 1 pathway, increasing expression of cellular antioxidant enzymes such as superoxide dismutase (SOD), catalase (CAT), and glutathione peroxidase (GSH Px), thereby strengthening defenses against oxidative stress [10,38,39]. Caffeic and ferulic acids are likewise well known for their antioxidant capacities [10,39], and have been reported in other *Eryngium* species [10,40,41]. β Sitosterol, present in plants and relevant in the human diet for its antioxidant effects, stimulates SOD and GPX activity and reduces catalase activity, favoring the glutathione-dependent pathway and supporting its therapeutic potential [23,42].

α-Glucosidase catalyzes the release of glucose from complex carbohydrates, contributing to postprandial glycemia. Dysregulation of this enzymatic activity can promote the development of type 2 diabetes; therefore, its inhibition is an effective strategy to blunt postprandial peaks, as exemplified by competitive inhibitors such as acarbose [43]. In this context, leaf and root extracts of *Eryngium* (EB, EH, and EM) exhibited effective α-glucosidase inhibition, suggesting a hypoglycemic potential associated with their metabolites. Previous studies on aerial parts of *Eryngium* species reported inhibitions of 55.4%, 39.5%, 33.7%, and 27.7% at 1000, 500, 250, and 125 μg/mL, respectively, for methanolic extracts of *E. caeruleum*, with an IC_50_ of 855 μg/mL [44]. Aqueous extracts of *E. cymosum* showed 32% inhibition of α-glucosidase [45]. These values are lower than those obtained in the present study. Conversely, hydroethanolic (70% ethanol) extracts of *E. maritimum* evaluated at 10 mg/mL reported 63.1% inhibition against microbial α-glucosidase and 29.0% against mammalian α-glucosidase in root extracts; leaf extracts showed even higher inhibitions of 72.3% and 50.8%, respectively [32]. These results underscore the inhibitory potential of EB, EH, and EM relative to other previously studied species of the genus. Caffeic acid, a hydroxycinnamic compound, has been reported to mitigate type 2 diabetes by improving glucose uptake, insulin secretion, and antioxidant activity [46,47], catechin exhibited 58.8% inhibition of α-glucosidase activity, significantly exceeding the effect of acarbose (36.6% inhibition) [48]. These findings are consistent with prior reports indicating that phenolic acids and flavonoids inhibit α-glucosidase [49,50]. In *E. planum*, rutin (290.5 μg/mL), caffeic acid (5.6 μg/mL), and ferulic acid (7.0 μg/mL) were identified as primary contributors to inhibition of enzymatic activity relevant to type 2 diabetes management [51]. In *E. bornmuelleri*, leaf extracts containing rutin, ferulic acid, and caffeic acid have shown inhibitory activity against digestive enzymes such as α-glucosidase [41]. β Sitosterol has been reported to inhibit α-glucosidase by 65.4% at 100 μg/mL and to significantly increase glucose uptake (28% at 100 μg/mL) in ethanolic extracts of two medicinal plants (*Cyperus rotundus* and *Tinospora cordifolia*) [52].

The above results demonstrate the potential of the three studied species, whose secondary metabolite content and antioxidant and antihyperglycemic activities support their possible application in traditional medicine; however, further phytochemical and biological investigations of these species are required.

## 4. Materials and Methods

Ethanol, methanol, dichloromethane, DPPH• (1,1-diphenyl-2-picrylhydrazyl), ABTS•+ (2,2′-azino-bis(3-ethylbenzothiazoline-6-sulfonic acid) diammonium salt), Trolox ((±)-6-hydroxy-2,5,7,8-tetramethylchromane-2-carboxylic acid), natural products reagent (diphenylborinic acid aminoethyl ester), anisaldehyde, phenolic standards and flavonoids (p coumaric acid, gallic acid, caffeic acid, ferulic acid, rutin, catechin, quercetin), and phytosterols (β sitosterol, campesterol), as well as dimethyl sulfoxide (DMSO), α glucosidase, p nitrophenyl α D glucopyranoside (p NPG), and acarbose were purchased from Sigma Aldrich (St. Louis, MO, USA). Silica gel HPTLC plates (60 F254, 20 × 10 cm) were supplied by Merck (Darmstadt, Germany). Mobile phase solvents (ethyl acetate, acetic acid, toluene, and formic acid) were obtained from Productos Químicos Monterrey Fermont (Monterrey, Mexico).

### 4.1. Plant Samples

Whole plants of EB and EM were collected in Los Tábanos, Jiquilpan de Juárez (19°58′59″ N, 102°49′51″ W), and EH in Marcos Castellano (20°03′37″ N, 102°50′06″ W), both in the state of Michoacán, Mexico. Specimens were placed in plastic bags and transported in a cooler to the laboratory of the Centro Interdisciplinario de Investigación para el Desarrollo Integral Regional, Instituto Politécnico Nacional (CIIDIR--IPN), Michoacán Unit. Plant identification was performed by M.C. Ignacio García Ruíz, curator of the CIMI herbarium at CIIDIR IPN, where the voucher specimens EB (13,691), EH (13,692), and EM (12,693) are deposited.

### 4.2. Preparation of EB, EH, and EM Extracts

Plants were rinsed with tap water and dried on paper towels. Roots and leaves were separated and lyophilized (FreeZone 12 freeze dryer, Labconco, Kansas City, MO, USA). Lyophilized tissues were ground to a fine powder and transferred in plastic bags wrapped in aluminum foil to the Centro de Desarrollo de Productos Bióticos, Instituto Politécnico Nacional (CEPROBI IPN) for further processing.

For metabolite extraction, the following solvent systems were used: ethanol–water (1:1, *v*/*v*), ethanol–water (7:3, *v*/*v*), absolute ethanol, absolute methanol, and dichloromethane. Extractions were performed by mixing 4 mg of lyophilized sample (leaf or root) with 4 mL of solvent (final concentration 1 mg/mL) and sonicating (Cole Parmer, DENTSPLY EQUIPAR, S.A. de C.V., Mexico City, Mexico) for 1 h at room temperature (22 ± 2 °C). Extracts were filtered through 0.45 µm PTFE syringe filters (13 mm, hydrophobic) and stored at 4 °C. For biological assays, leaf and root extracts in ethanol–water (7:3, *v*/*v*) at 1 mg/mL were used.

### 4.3. Determination and Quantification of Metabolites by High-Performance Thin-Layer Chromatography (HPTLC)

For metabolite identification and quantification, samples were applied in triplicate (1 mg/mL) onto 20 × 10 cm TLC plates coated with silica gel 60 F254 (Merck^®^, Darmstadt, Germany). A total of 18 bands were spotted, each positioned 8.0 mm from the lower edge, with an application length of 6.0 mm and a spacing of 10.0 mm between bands, starting from a left lateral position of 14.0 mm, using a sample applicator (LINOMAT 5, CAMAG, Muttenz, Switzerland). Plates were developed with 10 mL of solvent mixture following the procedure of Thiem et al. [30], using an automated development chamber (ADC 2, CAMAG, Muttenz, Switzerland) at 47% relative humidity (RH). Derivatization was performed by immersing the plates in a solution containing diphenylborinic acid aminoethyl ester (natural products reagent) and anisaldehyde for 1 s. After derivatization, plates were dried for 3 min at room temperature (22 ± 2 °C) and subsequently heated for 5 min at 100 °C using a TLC Plate Heater 3 (CAMAG, Muttenz, Switzerland). Images of each plate were captured using a TLC visualizer (CAMAG, Muttenz, Switzerland) under visible light and ultraviolet light at 254 nm and 366 nm, respectively.

Flavonoids (rutin, catechin, quercetin), phenolic compounds (p coumaric acid, gallic acid, caffeic acid, ferulic acid), and phytosterols (β sitosterol and campesterol) were used as standards, as these compounds are commonly present in plants. Aliquots of 5 μL from each extract and 3 μL of each standard (1 mg/mL) were analyzed.

The plates were analyzed using a TLC scanner (CAMAG, Muttenz, Switzerland). The scanner employed UV light at wavelengths of 220, 254, 330, 366, and 600 nm to detect compounds present in the samples. The resulting densitograms displayed peaks corresponding to each metabolite, enabling their identification and quantification. Additionally, the UV VIS absorption spectra of the target compounds were examined to confirm their identities. Spectral data were compared with reference spectra of known standards, providing further validation through the calculation of percentage similarity between sample and standard spectra. The results were processed using VisionCATS software version 3.2.23346.3 (CAMAG, Muttenz, Switzerland).

The concentrations of rutin, catechin, gallic acid, caffeic acid, ferulic acid, and β sitosterol were quantified by estimating the height of specific peaks using dedicated calibration curves: rutin (y = 5.694 × 10^−9^ x, R = 0.9927), catechin (y = 2.605 × 10^−7^ x, R = 0.9961), gallic acid (y = 8.391 × 10^−9^ x, R = 0.9930), caffeic acid (y = 1.224 × 10^−8^ x, R = 0.9942), ferulic acid (y = 2.6665 × 10^−7^ x, R = 0.9908), and β sitosterol (y = 1.344 × 10^−9^ x, R = 0.9946). For each analysis, variable volumes (0.5, 1, 1.5, 2, 2.5, 3 μL; equivalent to 0.5, 1, 1.5, 2, 2.5, 3 μg) of standard solutions (1 mg/mL in ethanol) were applied along with triplicate samples from each plant organ.

For rutin, the plate was eluted with a mixture of ethyl acetate, acetic acid, and water (8:1:1, *v*/*v*/*v*), and visualized using natural product reagents. In contrast, catechin, gallic acid, caffeic acid, ferulic acid, and β sitosterol were eluted with toluene, ethyl acetate, and formic acid (6:4:0.3, *v*/*v*/*v*). The first four compounds were visualized with natural product reagents, while β sitosterol was detected using anisaldehyde reagent. The method was validated for instrument performance, linearity, accuracy, repeatability, specificity, and linear response. Results were expressed in μg/mL of the detected metabolites.

### 4.4. Antioxidant Activity by ABTS•+ and DPPH• Microdulution Method

#### 4.4.1. Antioxidant Activity via ABTS•+ Assay

The antioxidant activity of the extracts was evaluated using the ABTS•+ method with minor modifications [53]. This technique provides a comprehensive assessment of the total antioxidant capacity. The ABTS•+ radical was generated by oxidation with potassium persulfate, and the solution was incubated in the dark at 4 °C for 16 h. The resulting solution was then diluted with double-distilled water until reaching an absorbance of 0.70 ± 0.01, measured using a spectrophotometer (SmartReader™ 96-T, MR9600-T, Accuris Instruments, Benchmark Scientific Inc., Edison, NJ, USA).

In a 96-well microplate, 20 μL of extract was added to each well, followed by 280 μL of ABTS•+ solution. The plate was incubated in the dark at room temperature (22 °C ± 2) for 6 min. After incubation, the absorbance of the sample and the blank was measured at 734 nm using the spectrophotometer (SmartReader™ 96-T, MR9600-T, Accuris Instruments, Benchmark Scientific Inc., Edison, NJ, USA). The antioxidant capacity was expressed as micromoles of Trolox equivalents per gram of dry weight (μmol TE/g DW). A calibration curve was constructed using five Trolox standards ranging from 0.00 to 0.60 μmol (μmol TE = −1.0377 × A_734_ + 0.9141, R^2^ = 0.9972). To determine the IC_50_ against the ABTS•+ radical, three concentrations (30, 20, and 10 μg/mL) were tested, and the percentage inhibition was calculated using Equation (1). All measurements were performed in triplicate to ensure reproducibility.

Inhibition (%) = 1 − (Sample absorbance)/(Blank absorbance) × 100(1)

#### 4.4.2. Antioxidant Activity via the DPPH• Assay

The antioxidant activity of EB, EH, and EM extracts was quantified using the DPPH• assay following the methodology described by Bernal et al. [53]. The DPPH• method primarily assesses the activity of non-polar antioxidants. In 96-well microplates, 20 μL of extract and 200 μL of DPPH• solution (150 mM), were added, and the mixture was incubated in the dark for 30 min. After incubation, the decrease in absorbance was measured at 515 nm using a spectrophotometer (SmartReader™ 96-T, MR9600-T, Accuris Instruments, Benchmark Scientific Inc., Edison, NJ, USA). The antioxidant capacity was expressed as micromoles of Trolox equivalents per gram of dry weight (μM TE/g DW), using a linear calibration curve based on Trolox standards (μM TE = −0.720 × A_515_ + 0.302, R^2^ = 0.9879). Each sample was analyzed in triplicate. The IC_50_ against the ABTS•+ radical was calculated from three concentrations (40, 30, 20, 10, and 0 μg/mL), and the percentage of inhibition was determined using Equation (1). All measurements were performed in triplicate to ensure reproducibility.

### 4.5. Antihyperglycemic Activity

The inhibition of α-glucosidase was determined using a microplate spectrophotometric method with slight modifications [43]. In a 96-well microplate, 170 μL of phosphate buffer (5 mM, pH 6.8) was added, followed by 10 μL of sample and 10 μL of α-glucosidase (0.4 U/mL). The mixture was incubated for 10 min at 37 °C. Subsequently, 10 μL of substrate (p NPG, 0.5 mM) was added to each well, and the plates were incubated again at 37 °C for 30 min. Absorbance was measured at 405 nm using a spectrophotometer (Multiskan FC, ThermoFisher^®^, Vantaa, Helsinki, Finland). DMSO served as the negative control, while acarbose (5 mg/mL) was used as the positive control. The IC_50_ was calculated based on the α-glucosidase response to three extract concentrations (40, 30, 20, 10, and 0 μg/mL). The percentage of inhibition at 100 μg/mL was determined using Equation (1). All measurements were performed in triplicate for each sample to ensure reproducibility

### 4.6. Statistical Analysis

A one-way analysis of variance (ANOVA) was performed for each organ within each species, along with a two-way ANOVA to compare among species, using a factorial experimental design: species (3 levels) and compounds (6 levels). Mean separation was conducted using Tukey’s test (*p* < 0.05). The statistical analyses were carried out using R Studio version 4.0.3. The half-maximal inhibitory concentration (IC_50_) was calculated using the Quest Graph™ IC_50_ Calculator, a software tool from AAT Bioquest (https://www.aatbio.com/tools/ic50-calculator, accessed on 28 June 2025).

## 5. Conclusions

The three evaluated *Eryngium* species were shown to be rich sources of secondary metabolites. Rutin, catechin, gallic acid, caffeic acid, ferulic acid, and β sitosterol were successfully detected, with higher concentrations observed in leaf tissues compared to roots. Ethanolic–water extracts (7:3, *v*/*v*) from EB, EH, and EM exhibited notable antioxidant capacity in ABTS•+ and DPPH• assays, along with effective inhibition of α-glucosidase activity. These findings suggest their potential in traditional Mexican medicine, where they are used to treat conditions such as diabetes, digestive disorders, and inflammation.

Based on these results, the three species demonstrate promising potential as sources of metabolites with notable biological activities. However, further detailed studies are necessary to validate their efficacy within traditional medicinal applications.

## Figures and Tables

**Table 1:** Rutin and catechin content in plant extracts of EB, EH, and EM.

Species	Rutin		Catechin	
	Leaf	Root	Leaf	Root
*E. beecheyanum*	241.3 ± 6.3 a	ND	94.2 ± 0.5 b	61.3 ± 1.4 a
*E. heterophyllum*	189.1 ± 5.3 b	ND	88.3 ± 2.2 c	41.8 ± 2.4 b
*E. mexiae*	NQ	ND	137.3 ± 0.3 a	64.4 ± 2.0 a

Results expressed as µg/mL (mean ± SD). ANOVA was performed; different letters within each column indicate significant differences (Tukey’s test, *p* ≤ 0.05). NQ = not quantifiable; ND = not detected.

**Table 2:** Ferulic acid, gallic acid, and caffeic acid content in plant extracts of EB, EH, and EM.

Species	Ferulic Acid		Gallic Acid		Caffeic Acid	
	Leaf	Root	Leaf	Root	Leaf	Root
*E. beecheyanum*	71.1 ± 1.9 b	NQ	32.5 ± 1.6 a	NQ	71.3 ± 1.9 a	NQ
*E. heterophyllum*	105.5 ± 1.3 a	NQ	31.6 ± 1.0 a	NQ	37.6 ± 1.3 b	7.4 ± 0.4 b
*E. mexiae*	108.7 ± 1.7 a	NQ	31. 8 ± 1.2 a	11.3 ± 0.8 a	68.5 ± 1.7 a	105.3 ± 0.8 a

Results expressed as µg/mL (mean ± SD). ANOVA was performed; different letters within each column indicate significant differences (Tukey’s test, *p* ≤ 0.05). NQ = not quantifiable.

**Table 3:** *β*-sitosterol content in dichloromethane extracts of EB, EH, and EM.

Species	*β*-Sitosterol	
	Leaf	Root
*E. beecheyanum*	197.5 ± 4.5 b	394.7 ± 6.5 a
*E. heterophyllum*	315.9 ± 2.3 a	NQ
*E. mexiae*	173.9 ± 5.8 c	93.0 ± 1.4 b

Results expressed as µg/mL (mean ± SD). ANOVA was performed; different letters within each column indicate significant differences (Tukey’s test, *p* ≤ 0.05). NQ = not quantifiable.

**Table 4:** Antioxidant activity of leaf extracts from EB, EH, and EM.

Specie	ABTS ●+ ^1^	Inhibition % ^2^	IC_50_ ^3^	DPPH ● ^1^	Inhibition % ^2^	IC_50_^3^
*E. beecheyanum*	220.0 ± 5.5 a	71.3	20.0	101.2 ± 3.0 a	85.5	19.7
*E. heterophyllum*	187.0 ± 2.7 b	64.4	21.5	46.8 ± 2.8 b	64.9	20.1
*E. mexiae*	215.0 ± 6.2 a	69.5	20.7	52.6 ± 1.1 b	67.4	22.4

Values are mean ± SD. ANOVA and Tukey’s test were performed (*n* = 9, *p* < 0.05); different letters within a column indicate significant differences. ^1^ µM TE/g PS; ^2^ Response observed at an extract concentration of 30 µg/mL; ^3^ µg/mL.

**Table 5:** Antioxidant activity of root extracts from EB, EH, and EM.

Specie	ABTS ●+ ^1^	Inhibition % ^2^	IC_50_ ^3^	DPPH ● ^1^	Inhibition % ^2^	IC_50_ ^3^
*E. beecheyanum*	28.6 ± 3.0 c	10.0	23.2	58.0 ± 1.4 b	68.4	23.1
*E. heterophyllum*	266 ± 3.1 a	89.4	21.8	84.3 ± 3.0 a	78.2	20.7
*E. mexiae*	64.4 ± 5.0 b	21.4	22.4	88.6 ± 1.4 a	81.4	18.0

Values are mean ± SD. ANOVA and Tukey’s test were performed (*n* = 9, *p* < 0.05); different letters within a column indicate significant differences. ^1^ µM TE/g PS; ^2^ Response observed at an extract concentration of 30 µg/mL; ^3^ µg/mL.

**Table 6:** Inhibition of α-glucosidase by extracts of EB, EH, and EM.

Specie	Inhibition in leaf (%) ^1^	IC_50_^2^	Inhibition in root (%) ^1^	IC_50_ ^2^
*E. beecheyanum*	37.4 ± 2.1 c	7.1	41.7 ± 1.6 b	5.8
*E. heterophyllum*	26.8 ± 2.9 d	9.6	42.8 ± 2.0 b	5.8
*E. mexiae*	49.1 ± 1.2 b	5.1	42.3 ± 1.6 b	5.9
Acarbose (CTL+)	80.4 ± 1.3 a	2.7	80.4 ± 1.3 a	2.7

Values are mean ± SD. ANOVA and Tukey’s test were performed by organ (leaf, root) (*n* = 9, *p* < 0.05); different letters within a column indicate significant differences. ^1^ Response observed at an extract concentration of 10 µg/mL; ^2^ µg/mL.

## Data Availability

In this work, original data was created and there is no restriction on its use due to any privacy issue.
